# Supplementary material for: Implementation and effectiveness outcomes of Community Health Advocacy Teams to improve long-lasting insecticide net distribution and use in six districts in Ghana: A one-group pre-post-test study
Source: PLOS Glob Public Health. 2024 Apr 1;4(4):e0002123. doi: 10.1371/journal.pgph.0002123 (PMC10984411; doi:10.1371/journal.pgph.0002123)
Supplement: Attachment — Submitted filename: Response to Reviewers 30.12.23.docx [file pgph.0002123.s002.docx]

December 30th ,2023

The Academic Editor
PLOS Global Public Health

Dear Sir or Madam:

**ADDRESSED REVIEW COMMENTS**

Thank you for the constructive feedback provided on the manuscript entitled ***“Implementation and effectiveness outcomes of Community Health Advocacy Teams to improve long-lasting insecticide net distribution and use in six districts in Ghana: a one-group pre-post-test study”***. Below are comments made by reviewers, as well as point-by-point responses indicating how and where these have been addressed throughout the manuscript.

| **S/N** | **REVIEW COMMENT** | **RESPONSES** |
| --- | --- | --- |
| **A.** | **Editor’s Comments** |  |
| 1. | We noticed you have some minor occurrence of overlapping text with the following previous publication(s), which needs to be addressed:  <https://www.frontiersin.org/articles/10.3389/frhs.2022.1102328/full>  In your revision ensure you cite all your sources (including your own works), and quote or rephrase any duplicated text outside the methods section. Further consideration is dependent on these concerns being addressed.""" | All the manuscripts have been cited accordingly. There is not direct quote from previously published work of the authors |
| 2. | Please include a complete copy of PLOS’ questionnaire on inclusivity in global research in your revised manuscript. Our policy for research in this area aims to improve transparency in the reporting of research performed outside of researchers’ own country or community. The policy applies to researchers who have travelled to a different country to conduct research, research with Indigenous populations or their lands, and research on cultural artefacts. The questionnaire can also be requested at the journal’s discretion for any other submissions, even if these conditions are not met.  Please find more information on the policy and a link to download a blank copy of the questionnaire here: [https://journals.plos.org/globalpublichealth/s/best-practices-in-research-reporting](https://journals.plos.org/globalpublichealth/s/best-practices-in-research-reporting). Please upload a completed version of your questionnaire as Supporting Information when you resubmit your manuscript. | We have completed the PLOS questionnaire and uploaded as a supplementary file |
| B. | **Reviewer #1** |  |
| 3. | **General comments**: It seems that certain sections and paragraphs might require revision to enhance readability. There are specific ideas that could benefit from improved linkage, ensuring greater clarity and fluency in the narrative. For instance, there are certain sentences in the 1st paragraph of the background section appears repetitive in their content. | The first paragraph of the background section has been revised to improved linkage, ensuring greater clarity and fluency in the narrative (Page 4) |
| 4. | The background should contextualise the study presented and should serve to prepare the reader understand the research gaps the paper aims to address. In this vein, the authors state that “more social, cultural, and behavioural research is needed to understand how local knowledge of transmission, diagnosis, treatment, and prevention influences the utilisation of ITN interventions” and “in order to create community-based malaria control programmes that are sustainable and encourage behaviour change as well as the adoption of new concepts and technologies, it is helpful to understand local knowledge”. While this argument is legitimate and supported by previous projects and accumulated evidence (and with which I agree), the study does not seem to cover these identified gaps, creating confusion for the reader… I suggest rephrasing this important idea to integrate it better into the background section's narrative flow | This section has been revised to include some barriers identified in previous studies such as lack of limited social and behaviour change communication (SBCC) activities, lack of continuous malaria education, knowledge gap on malaria prevention, which serves as a basis for more social, cultural, and behavioural research to be conducted to understand how local knowledge of transmission, diagnosis, treatment, and prevention influences the utilisation of ITN interventions and to develop culturally appropriate and sustainable intervention (CHAT) that will contribute to LLIN use and ownership and malaria prevention in Ghana (Pages 5 and 6) |
| 5. | Another aspect that might require revision is the presentation of the CHAT intervention itself. Certain information appears to be missing (for instance, its origin: Who initiated this initiative? Is it part of a broader project? Is it affiliated with NMEP?).  Additionally, providing more details on the co-creation process of the CHAT intervention would be highly appreciated | Revision has been made to description of the CHAT intervention to include; it was an initiative by the study team from the University of Ghana School of Public Health in collaboration with the NMEP.  Also, additional details have been provided on the co-creation process of the CHAT intervention (Pages 6 and 7) |
| 6. | The authors state that they utilized the RE-AIM model, which apparently informed the selection and evaluation of effectiveness and implementation outcomes (i.e., acceptability, appropriateness, feasibility). However, given that the RE-AIM model comprises five dimensions pertinent to public health program planning and evaluation, none of which specifically address the selected implementation outcomes of this study (at least, not using the same terms), it would be helpful to provide details on how the model was employed and how it guided the assessment of short-term effectiveness and implementation outcomes. Specifically, clarifying the relationship between this conceptual model and the ultimately chosen outcomes is essential | More details have been provided on the use of the RE-AIM model as the basis for determining and assessing the implementation outcomes and short-term effectiveness of our study outcomes (Pages 8 and 9) |
| 7. | There are additional concerns regarding the methodology. Firstly, the authors state that this study follows a one-group pre-post design. I assume this means the same group of participants was involved in both phases of the study; however, the figures presented in Table 1 raise some doubts in this regard. I would appreciate clarification from the authors on this matter. | This has been clarified. There were some few differences between the baseline and endline figures observed (i.e., Birim South and Achiase) and this was due to the unavailability of some participants (n=2) who participated in the baseline hence any other available member of the same household who met the selection criteria were involved. (Pages 12-13) |
| 8. | Secondly, unlike the short-term effectiveness, there are no examples of the questions addressing acceptability, appropriateness, and feasibility. Given the complexity of defining and measuring these constructs, it would be valuable for the authors to provide some specific details. This will also allow to fully understand the results presented in the various tables. | We have provided more details. We adopted the questions for the RE-AIM model and the acceptability framework by Sekhon and colleague (page 9) |
| 9. | In the discussion section, the authors address the crucial topic of trust-building, a key aspect often emphasized when evaluating the acceptability of a public health intervention at the community level. However, the connection between the collected data and the topic of trust is not sufficiently clear. I recommend that the authors elaborate more on this argument to enhance the understanding of how trust is implicated in their findings | This has been elaborated to include the engagement of stakeholders and community members throughout all phases of this study have contributed to trust building. Hence, the high acceptability observed here can be attributed to the approach used in this study, such as the use of SBCC, community mobilisation, and engagement of community stakeholders and community members throughout the intervention development and implementation processes. (Pages 15 and 16) |
| 10. | **Background:**  Line 71: Is the reference 1 cited correctly referred in the bibliography section? In case the authors wanted to cite the World Malaria Report 2022, the reference should be revised and corrected accordingly | This has been revised accordingly |
| 11. | Line 147-148: The acronym for the National Malaria Elimination Programme (NMEP) has already been used earlier in the text. There’s no need to use the long name again. I recommend the authors revise this throughout the text since there are other instances. | This change has been made. (Page 6) |
| 12. | **Methods:**  Line 239: The 1st paragraph introducing the subsection on data collection appears to be confusing at this point in the text. I suggest moving it up to the introduction section, where the objectives of the study are being presented. This placement would provide better context and clarity for the readers. | This paragraph has been moved to the introduction section (Pages 7 and 8) |
| 13. | **Discussion**:  Lines 353-356: I’m not sure if the results provided helps support this statement. Can you elaborate more on this? | Some additional sentence has been introduced for better comprehension. Thus, the engagement of stakeholders and community members throughout all phases of this study have contributed to trust building. Hence, the high acceptability observed here can be attributed to the approach used in this study, such as the use of SBCC, community mobilisation, and engagement of community stakeholders and community members throughout the intervention development and implementation processes of this study (Pages 15 and 160 |
| 14. | Lines 394: The exact same sentence has been used earlier in the text. While I understand that the idea is being reintroduced, I recommend rephrasing it to create a more favorable impression on the reader. | This has been rephrased as suggested |
|  | Reviewer #2 |  |
| 15. | **Major**:  The specifics of the CHAT intervention are not presented. Put simply, what did they actually do during the study period? How many meetings were there? How many household visits? There is no description of the activities conducted. | What the CHAT did during the study and the activities conducted have been indicated in the paper  Page-7; *The CHAT sensitizes the community on the proper use and maintenance of LLINs, supports with the management of LLINs logistics during distribution exercises and accountability, and assists other community-based health campaigns [13]. CHAT use community-based strategies such as house-to-house, community durbars, use community information centres and school health platforms to promote the use of ITNs. In addition, CHAT members collaborate with district health management team and other civil society groups to provide voluntary campaigns on LLINs use. The teams officially started their activities in the community in November 2021*).  Also, meeting days of the CHAT members has been added (page 7- *The CHAT members meetings are convened quarterly, preferably by a CHO).* |
| 16. | There are standard WHO definitions of LLIN coverage measures including ownership, access, target coverage (1 LLIN per 2 HH members) and use, that are not referenced here and would have made for a more robust analysis. For example, we people not sleeping under LLINs because they did not have any LLINs, or they did not have enough LLINs for everyone in the household, or they had enough, but chose not to? Each is very different. | As this study was context-based (Ghana) to develop a culturally appropriate intervention, data and references used in relation to LLIN use and ownership were based on reports/data/information from the National Malaria Elimination Programme in Ghana, which also uses standard definitions from WHO. Hence these were the measures/definitions used throughout the study. |
| 17. | The power calculation is not clearly described. What does a "50% anticipated use of mass LLIN distribution" refer to? A difference between pre- and post-surveys? | The power refers to difference between baseline and endline use. This addition can seen on page 10. |
| 18. | Willingness to pay analysis is a discrete field of economics and requires much more than just a binary yes/no response. The results presented here don't truly capture that complexity and likely suffer from a social desirability bias | Thank for the comment. We adopted a question from Ghana Malaria Indicator Survey |
| 19. | **Min**or:  Current accepted term is long-lasting insecticidal nets (LLINs), not insecticide treated nets | This has been corrected throughout the paper. |
| 20. | Authors go back and forth between LLIN and ITN | All ITN has been revised to LLINs throughout the paper |
| 21. | What does the proportion in each district mean? For example, Line 218. Is 90% and 75% the PfPR? If so that is an astounding level of transmission? | A sentence has been introduced to make it clearer; they are malaria prevalence rates recorded in the selected districts (Page 9) |
| 22. | Increasement (Line 332) should be increase | This correction has been made (Page 14) |

Thanks again for your kind review.

Yours Sincerely,

Dr. Philip Teg-Nefaah Tabong
