# Supplementary material for: Implementation and effectiveness outcomes of Community Health Advocacy Teams to improve long-lasting insecticide net distribution and use in six districts in Ghana: A one-group pre-post-test study
Source: PLOS Glob Public Health. 2024 Apr 1;4(4):e0002123. doi: 10.1371/journal.pgph.0002123 (PMC10984411; doi:10.1371/journal.pgph.0002123)
Supplement: Attachment — Submitted filename: Response to Reviewers 27.2.24.docx [file pgph.0002123.s003.docx]

December 30th ,2023

The Academic Editor
PLOS Global Public Health

Dear Madam,

**ADDRESSED REVIEW COMMENTS**

Thank you for the constructive feedback provided on the manuscript entitled ***“Implementation and effectiveness outcomes of Community Health Advocacy Teams to improve long-lasting insecticide net distribution and use in six districts in Ghana: a one-group pre-post-test study”***. Below are comments made by reviewers, as well as point-by-point responses indicating how and where these have been addressed throughout the manuscript.

| **S/N** | **REVIEW COMMENT** | **RESPONSES** |
| --- | --- | --- |
| **A.** | **Journal Requirements** |  |
| 1. | Please review your reference list to ensure that it is complete and correct. If you have cited papers that have been retracted, please include the rationale for doing so in the manuscript text, or remove these references and replace them with relevant current references. Any changes to the reference list should be mentioned in the rebuttal letter that accompanies your revised manuscript. If you need to cite a retracted article, indicate the article’s retracted status in the References list and also include a citation and full reference for the retraction notice. | Sall references are correct. No reference has been retracted |
| 2. | We have noticed that you have uploaded Supporting Information files, but you have not included a list of legends. Please add a full list of legends for your Supporting Information files after the references list. | This has been provided |
| B. | **Editors comments** |  |
| 3. | You mention several times the gap between LLIN access and LLIN use, suggesting that this is primarily a behavioral challenge. However, the indicator you have used to define access is a household with at least one LLIN, but where the household has more than 2 members this may not be "sufficient" nets for everyone to be able to sleep under a net. I suggest that this difference between having access to any net in a household and having sufficient nets is discussed at some point. You may also consider including the indicator of whether a household has at least one LLIN for every two members as an indicator of sufficient nets. | This has been clarified on page 4 line 105 |
| 4. | In the description of the study area you refer to the selected district has having highest prevalence, with a reference to the Ghana HMIS. However, routine HMIS data does not usually report prevalence since it is not a cross-sectional survey. Either this reference should be corrected to the MIS, or perhaps the actual indicator from HMIS used for selection of districts with incidence, or maybe test positivity rate among patients attending health facilities. | We cross checked that with NMEP and have revised the statement to malaria positivity rate (page 8 lines 234/235 |
| 5. | I would like to recommend including in the discussion a few sentences exploring whether the observed changes in your effectiveness outcomes are due entirely to the CHAT process, or partly a result of the point mass distribution occurring together with the CHAT. An alternative to this pre-post study design could have been to also conduct surveys in areas where PMD is taking place but without the CHAT component, allowing you to perform a difference in differences analysis and strengthen the plausibility that changes in your outcome indicators are attributable to the CHAT intervention. | We have clarified this (page 16) and added it as limitation of the study (page 17) |

Thanks again for your kind review.

Yours Sincerely,

Dr. Philip Teg-Nefaah Tabong
